# Gene expression profiling of 1200 pancreatic ductal adenocarcinoma reveals novel subtypes

**DOI:** 10.1186/s12885-018-4546-8

**Published:** 2018-05-29

**Authors:** Lan Zhao, Hongya Zhao, Hong Yan

**Affiliations:** Department of Electronic Engineering, City University of Hong Kong, 83 Tat Chee Ave, Kowloon Tong, Hong Kong

**Keywords:** Pancreatic ductal adenocarcinoma, Heterogeneity, Biclustering, Subtype, Deep learning, Biomarkers

## Abstract

**Background:**

Pancreatic ductal adenocarcinoma (PDAC) is the fourth leading cause of cancer related death in the world with a five-year survival rate of less than 5%. Not all PDAC are the same, because there exist intra-tumoral heterogeneity between PDAC, which poses a great challenge to personalized treatments for PDAC.

**Methods:**

To dissect the molecular heterogeneity of PDAC, we performed a retrospective meta-analysis on whole transcriptome data from more than 1200 PDAC patients. Subtypes were identified based on non-negative matrix factorization (NMF) biclustering method. We used the gene set enrichment analysis (GSEA) and survival analysis to conduct the molecular and clinical characterization of the identified subtypes, respectively.

**Results:**

Six molecular and clinical distinct subtypes of PDAC: L1-L6, are identified and grouped into tumor-specific (L1, L2 and L6) and stroma-specific subtypes (L3, L4 and L5). For tumor-specific subtypes, L1 (~ 22%) has enriched carbohydrate metabolism-related gene sets and has intermediate survival. L2 (~ 22%) has the worst clinical outcomes, and is enriched for cell proliferation-related gene sets. About 23% patients can be classified into L6, which leads to intermediate survival and is enriched for lipid and protein metabolism-related gene sets. Stroma-specific subtypes may contain high non-epithelial contents such as collagen, immune and islet cells, respectively. For instance, L3 (~ 12%) has poor survival and is enriched for collagen-associated gene sets. L4 (~ 14%) is enriched for various immune-related gene sets and has relatively good survival. And L5 (~ 7%) has good clinical outcomes and is enriched for neurotransmitter and insulin secretion related gene sets. In the meantime, we identified 160 subtype-specific markers and built a deep learning-based classifier for PDAC. We also applied our classification system on validation datasets and observed much similar molecular and clinical characteristics between subtypes.

**Conclusions:**

Our study is the largest cohort of PDAC gene expression profiles investigated so far, which greatly increased the statistical power and provided more robust results. We identified six molecular and clinical distinct subtypes to describe a more complete picture of the PDAC heterogeneity. The 160 subtype-specific markers and a deep learning based classification system may be used to better stratify PDAC patients for personalized treatments.

**Electronic supplementary material:**

The online version of this article (10.1186/s12885-018-4546-8) contains supplementary material, which is available to authorized users.

## Background

The pancreas is both an exocrine and endocrine gland, playing important roles in the digestive and endocrine systems. There are two kinds of cells in the pancreas: exocrine cells and endocrine cells. When exocrine cells grow out of control, they may form pancreatic exocrine tumors. About 95% of pancreatic cancers can be classified into pancreatic exocrine tumors. One kind of pancreatic exocrine tumor called pancreatic ductal adenocarcinoma (PDAC) is the most common type, making up more than 85% of all pancreatic cancers. PDAC is the fourth leading cause of cancer related death in the world with a 5-year survival rate of only 5% [1]. Surgery is by far the most effective treatment strategy for PDAC, but less than 20% of PDAC patients have resectable tumors at the time of diagnosis [2, 3], with the improving 5-year survival rate after resection to 10–25% [4, 5]. The etiology of PDAC are poorly understood. However, several factors like cigarette smoking [6], family history of pancreatic cancer [7], diabetes [8] and chronic pancreatitis [9] are contributing factors for PDAC.

Like other malignancies, the intra-tumoral heterogeneity makes PDAC not a single disease, but a group of biologically and clinically distinct diseases [10, 11]. Thus, there is a great need to identify homogeneous groups which is an essential step towards personalized treatment of PDAC. Traditional classification of PDAC has been carried out by pathologists based on histologic appearance and phenotypic traits. However, in reality, tumors with similar morphological appearance may have very distinct molecular features and clinical outcomes [12, 13]. Recent advancements in genome wide molecular profiling may change these situations by providing an opportunity to investigate the tumor heterogeneity at the whole genome level. Gene expression profiling, one of the most commonly used molecular profiling approaches, is the measurement of the expression levels of thousands of genes simultaneously. And, microarray and RNA sequencing (RNA-Seq) are the two most used techniques. Gene expression profiling have allowed researchers to classify cancers into homogeneous groups with improved diagnosis [14–16] and correlated better with survival information than traditional classification of cancers [17]. Over the last few years, increasing molecular classification studies have been conducted in PDAC which proved that it can be classified into 2 to 4 subgroups [18–24]. However, these studies used tumor samples ranging from dozens to more than few hundreds as their discovery cohort. They may not fully represent the intra-tumoral heterogeneity and limit the ability to identify rare subtypes of PDAC.

Another concern in dissecting the tumor heterogeneity is the methods used in the identification process. Given a set of gene expression profiles, clustering, a machine learning technique, can be used to group data objects of similar characteristics together into distinct clusters without prior assignment (unsupervised classification). There are three kinds of clustering strategies [25]: first, gene-based clustering, which the genes are treated as the objects, while the samples are the features. Second, sample-based clustering which the samples are the objects and genes are the features. And third, biclustering (or subspace clustering) which capture clusters formed by a subset of genes across a subset of samples. The previous two strategies apply a global model to identify clusters. That is, each sample in a subtype is determined by the activity of all the genes. Similarly, each gene in a given gene cluster is defined using all the samples when performing the clustering analysis [26]. Since subsets of genes are active or silent only under certain experimental conditions, and behave almost independently under other conditions [26], the classification results are relatively poor when using the global model [27].

Only biclustering employ a local model to identify coherent patterns in an expression matrix. Instead of clustering gene and sample separately, biclustering allows simultaneous clustering of genes and samples [26]. Thus, biclustering has become a popular technique and lots of algorithms are proposed, such as distance-based [28, 29], factorization-based [30, 31] and geometric-based biclustering [32, 33]. Most biclustering algorithms [34–38] allow bi-clusters to have partially overlap, and some objects (samples or genes) may not belong to any bi-cluster at all [39, 40]. This character of biclustering, although useful in some instances [26], is not good for interpretation. Non-negative Matrix Factorization (NMF), a dimensionality reduction and factorization-based biclustering algorithm, aims to find groups of linear combination of metagenes representing local patterns in the expression matrix. NMF has been proven useful in many cancer subtyping studies [18, 20, 23, 41, 42] due to its easy interpretation and desired performances.

In our study, we focused on using NMF to extract biclusters from gene expression data, thus to describe and characterize the heterogeneity of PDAC. We overcame the sample shortage by combining different sources of PDAC into a single and large dataset. Specifically, we collected publically available PDAC gene expression profilings from 11 microarrays and 3 RNA-Seq datasets. In total, our study involves more than 1200 PDAC patients, and 796 of them were used as the discovery cohort. This is the largest cohort of PDAC gene expression profiles investigated so far, which greatly increased the statistical power and provided more robust results. We identified six molecular and clinical distinct subtypes, and provided a deep learning-based classification system for PDAC. Compared with previous studies [18–24], our study has several advantages. First, we included more PDAC cases to increase statistical reliability. Second, we selected genes as subtype-specific biomarkers directly from biclusters. Third, we identified six subtypes to provide and describe a more complete picture of the PDAC heterogeneity. Last but not least, we used deep learning to build a classification system for PDAC, which can be used to classify new patients. The classification model will be publicly available upon request.

## Methods

### Data curation and pre-processing

We searched multiple data repositories, including the International Cancer Genome Consortium (ICGC, www.icgc.org), the Cancer Genome Atlas (TCGA, http://cancergenome.nih.gov/), Gene Expression Omnibus (GEO, http://www.ncbi.nlm.nih.gov/geo/) and ArrayExpress (https://www.ebi.ac.uk/arrayexpress/) for available gene expression profiling datasets for PDAC. We came across altogether 14 datasets, which were listed below:

We collected 3 RNA-Seq datasets in our study, one from TCGA, and another two from ICGC and GSE79670. RNA-Seq datasets were pre-processed as follows: RSEM values of TCGA Pancreatic Adenocarcinoma mRNA-Seq were downloaded through TCGA2STAT R package [43], which contains 172 non-overlapping primary PDAC patients with detailed clinical information. Data were subsequently normalized using TMM (weighted trimmed mean of M-values) with the EdgeR package [44], and converted to counts per million (cpm) and log2 transformed. A filtering process was also performed to exclude the genes without at least 1 cpm in 20% of the samples. Raw counts data of GSE79670, which contains 51 primary PDAC patients, were downloaded from GEO and normalized in the same way as in the TCGA dataset. The third and the last RNA-Seq dataset can be downloaded either from ICGC under the identifier PACA-AU, or from the supplemental material in the corresponding publication [23]. We chose to download this dataset from the latter option and named this dataset as Bailey. This dataset contains normalized expression values (data were normalized in the same way as in the previously mentioned two RNA-Seq datasets) of 96 pancreatic cancer patients and 71 of them were PDAC. Only PDAC samples were retained for the following analysis.

There were also 11 microarray datasets in our study, which were listed below according to their sample size: MTAB-1791 (195 primary PDAC, Illumina WG6 BeadChip v3 array), ICGCarray (178 primary PDAC, Illumina HT12 v3 array), GSE71729 (145 primary PDAC, Agilent-014850 array), GSE62165 (118 primary PDAC, Affymetrix U219 array), GSE62452 (69 primary PDAC, Affymetrix 1.0 ST array), GSE57495 (63 primary PDAC, Rosetta/Merck Affymetrix 2.0 array), GSE60980 (49 primary PDAC, Agilent-028004 array), GSE77858 (46 primary PDAC, Agilent-012097 array), GSE55643 (45 primary PDAC, Agilent-014850 array), GSE15471 (39 primary PDAC, Affymetrix U133 Plus 2.0 array) and Collisson (27 primary PDAC, Affymetrix U133 Plus 2.0 array). Among them, ICGCarray originally contains 269 PDAC tissue and pancreatic cell lines samples. After removing cell lines, non-PDACs and metastatic tumors, 178 primary PDAC tumor samples were retained. Datasets used in our study can be found in Table 1.

**Table 1:** Datasets used in the study

DataSet	Sample Size	Platform	clinical Data	Note
ICGCarray	178	Illumina	Yes	Training set
TCGA	172	RNA-Seq	Yes	Training set
MTAB-1791	195	Illumina	No	Training set
GSE62165	118	Affymetrix	No	Training set
GSE60980	49	Agilent-028004	No	Training set
GSE15471	39	Affymetrix, plus2	No	Training set
GSE55643	45	Agilent-014850	No	Training set
Bailey	71	RNA-Seq	Yes	Validation set
GSE71729	145	Agilent-014850	Yes	Validation set
GSE57495	63	Rosetta/Merck	Yes	Validation set
GSE79670	51	RNA-Seq	Yes	Validation set
GSE62452	69	Affymetrix	Yes	Validation set
Collisson	27	Affymetrix, plus2	Yes	Validation set
GSE77858	46	Agilent-012097	No	Validation set

We downloaded raw counts, processed microarray data, and associated clinical information from public data repositories for each dataset. Counts data were pre-processed as mentioned above. Then, the gene expression profile on probe level (or Ensembl ID level) was converted into official gene symbol level. When multiple probe sets (or Ensembl IDs) were mapped to the same gene symbol, the probe sets (or Ensembl IDs) with the largest mean expression values across samples were kept. Only primary tumor samples were retained. Metastasis samples or treated patients samples were excluded from the analysis. Datasets without clinical information were used for training. Except for GSE77858 dataset, which without clinical information, and used as one of the validation dataset, because this dataset has relatively low variable genes (~ 42 variable genes). In order to determine whether the identified subtypes have distinct survival differences, we also included two large datasets from ICGC and TCGA, which contain detailed clinical information, as our training datasets as well. So in total, 7 independent datasets from 5 platforms, with 796 primary PDAC patients were used for training. The remaining 7 datasets with 472 primary PDAC patients, were either combined or independently used as the validation datasets. Datasets were combined by concatenating data matrices together, followed by using ComBat [45] to adjust the introduced batch effects. Additional file 1: Figure S1 shows the principal component analysis (PCA) before and after batch effect correction for training and validation datasets.

### Identification of PDAC subtypes

Before performing NMF, a filtering procedure was applied to remove genes with low variability across the samples in 7 dataset from the training cohorts, respectively. The idea is that higher variable genes are informative in the clustering process. Specifically, the median absolute deviation (MAD) value of each gene was calculated. If the value was less than 0.5, then that gene was excluded from the clustering analysis.

The filtering step resulted in 411 most variable genes that were kept for the clustering process. NMF R package [46] was used to perform clustering using the Brunet algorithm. We varied the number of clusters k from 2 to 10 and repeated the clustering process 30 times. The value of k that results in the maximum cophenetic correlation coefficient was chosen as the optimal number of clusters. Then we performed clustering 200 times with optimal k and random initialization to obtain the consensus matrix, sample labels and associated meta-genes.

### Generation of the PDAC classifier and classification

**Fig. 1:**
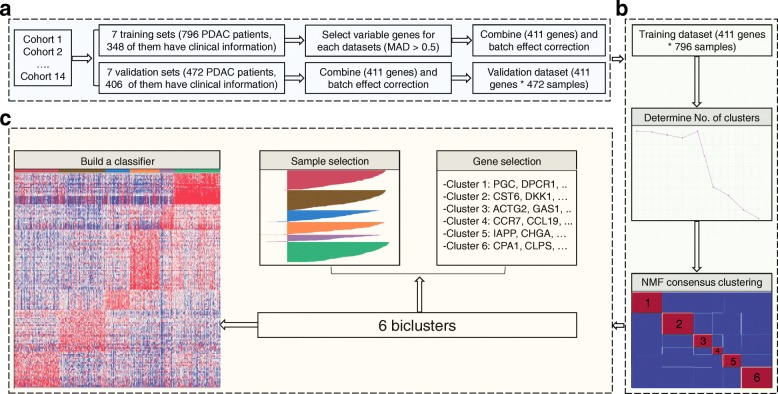
The flowchart of the classifier building process. **a** Data processing step. Fourteen datasets were collected and separated into training and validation datasets. Four hundred eleven most variable genes were then selected based on the median absolute deviation (MAD > 0.5), and were kept for the clustering process. **b** NMF clustering step. Six-cluster resulted the maximum cophenetic correlation coefficient was chosen as the optimal number of clusters. Then, NMF clustering were performed of 200 times with optimal number of clusters to obtain the consensus matrix. **c** Classifier building steps. A classifier was built on the most representative samples and most predictive genes for each cluster

A classifier was built on the most representative samples and most predictive genes for each cluster. Silhouette width [47] was computed to identify the most representative samples using the R package Cluster. Subtype specific genes were determined using the extractFeatures function in the NMF package [46], with the largest row feature scores. Then, SAM (Significance Analysis of Microarrays) [48] analysis was performed to filter out unstable genes between clusters. Figure 1 summarized the classifier building process.

We trained a deep learning model as the PDAC classifier using the H2O R package [49]. We split the training dataset into three parts when building the model: 60% for training, 20% for validation and the remaining 20% for testing. The parameters we used were as follows: TanhWithDropout activation, bernoulli distribution, and two hidden layers with 500 neurons each. The other parameters were set as default. The classification performance of the classifier was verified on the training and validation datasets.

### Gene set enrichment analysis (GSEA)

Before GSEA, we used the limma package [50] to calculate the fold changes of one subtype versus all other subtypes in the combined training dataset. For each subtype, more than 10, 000 genes fold change values were used as the input data in the GSEA analysis. In our study, GSEA was performed using the R package Piano [51], together with the version 6.0 annotated gene sets (H, C2 and C5) downloaded from the MsigDB database. We used the gene sets with the number of genes ranging from 10 to 500, 1, 000 permutations for gene sampling and 20 cpus to conduct the analysis. Significantly enriched gene sets (adjust *p*-value less than 0.05) were ranked according to consensus scores [51], top 10 representative gene sets with largest consensus scores were selected for each subtype, respectively, and used for heatmap visualization. Specifically, a data matrix was generated with rows defined by the selected gene sets, and columns by consensus scores for each subtype. Then, pheatmap R package was used for the heatmap visualization.

### Survival analysis

Clinical data were downloaded from associated published results. Median survival was estimated using the Kaplan–Meier method and the difference was tested using the log-rank test. *P*-values of less than 0.05 were considered statistically significant. We also applied Fisher’s exact test to investigate the relationships among subtype, tumor stage, tumor grade and other clinical information (Additional file 2: Table S1).

## Results

### NMF identifies six subtypes in PDAC

**Fig. 2:**
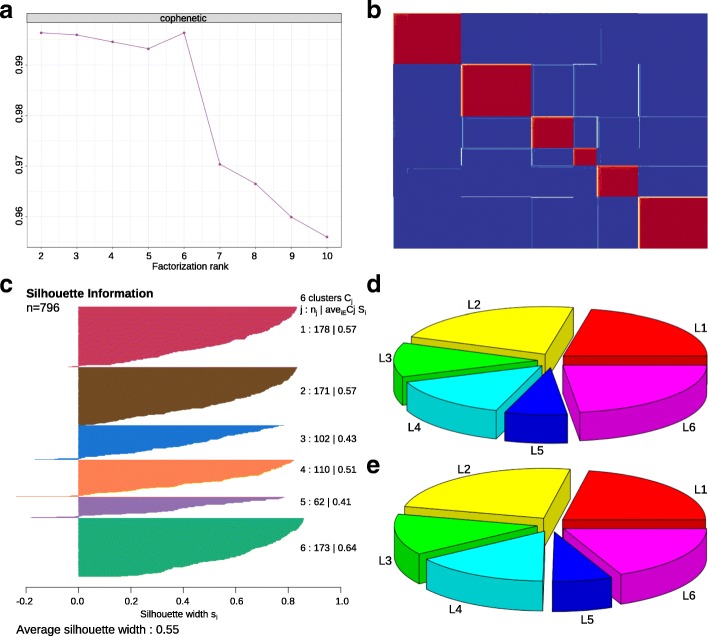
Classification of PDAC into 6 subtypes. **a** Unsupervised classification of PDAC using NMF. A peak cophenetic correlation was observed for *k* = 6 classes. **b** Consensus matrix for *k* = 6 is shown. **c** Silhouette information for *k* = 6 classes. **d** Patient distribution in the training dataset (*n* = 796). **e** Patient distribution in the merged validation dataset (*n* = 472)

We applied NMF to the merged training dataset (796 PDAC patients), and obtained 2 to 6 well-defined clusters (Additional file 3: Figure S2). Cophenetic correlation coefficients were calculated to determine the optimal number of clusters, and a peak was found at k = 6 (Fig. 2a). The consensus matrix heatmap contains sharp and crisp boundaries, which implies stable and robust clustering for the samples (Fig. 2b). Silhouette width analysis was subsequently performed to select the most representative samples within each cluster (Fig. 2c). The average silhouette width was 0.55 (range, from 0.41 to 0.64), indicating the robustness of the classification. A total number of 781 samples (~ 98%) with positive silhouette width were retained to build the classifier.

Next, 160 metagenes identified by NMF were selected as features (Table 2), together with 781 sample’s Z-score normalized data to build a deep learning classifier of PDAC. We used the H2O package to split the merged training dataset into three parts: internal training set (470 PDAC, 60%), internal validation set (152 PDAC, 20%) and internal test set (159 PDAC, 20%). The internal training set was used for building the model, the internal validation set for early stopping, and internal test set for testing the classification error. The classification errors on the internal training set and internal test set were 0.8 and 13%, respectively (Additional file 4: Table S2). The classifier can be used to classify all the 796 PDAC patients in the training dataset into six subtypes: L1 (174 patients, 21.9%), L2 (176 patients, 22.1%), L3 (93 patients, 11.7%), L4 (113 patients, 14.2%), L5 (56 patients, 7.0%) and L6 (184 patients, 23.1%) (Fig. 2d). We also did the classification with the combined validation dataset. Patients in this dataset can also be classified into six subtypes with a similar proportions of patients being distributed among subtypes (Fig. 2e). In addition, we found that there were 65 overlapped samples between our training and combined validation dataset. More specifically, 65 samples were overlapped between ICGCarray set (178 PDAC, microarray platform) and Bailey set (71 PDAC, RNA-Seq platform). We extracted the 65 predicted sample labels from these two cohorts and compared the similarities between them. Result shows that the two lists were similar, except that there were 17 samples with inconsistent classification results, which may be jointly caused by platform differences and the classification error of the classifier.

**Table 2:** Subtype specific gene lists

Tumor-specific subtypes			Stroma-specific subtypes		
L1	L2	L6	L3	L4	L5
AGR2	ADM	AKAP7	ACTG2	ADAMTS1	ABCC8
ALDOB	ANGPTL4	ALB	CDH11	C1orf162	ADAMTSL2
ANXA13	C19orf33	ANPEP	COL10A1	CCL2, 19, 21	C7
AQP5	CCNB2	AQP8	COL12A1	CCR7	CHGA
ARL14	CDH3	CEL	COL8A1	CD2, CD3D, CD6, CD8A, CD36, CD48, CD52, CD69, CD79B, CD163, CD247	COLEC11
C4BPB	CDKN2A	CLPS	COLEC12	CFD	CPE
CA2	COL7A1	CPA1	GAS1	CILP	F2RL2
CDCA7	CRABP2	CPB1	GREM1	CXCL9, 10, 12	FRZB
CTSE	CST6	CTRC	LRRC17	CXCR4	G6PC2
CYP3A5	DCBLD2	CTRL	MFAP5	EVI2B	IAPP
DMBT1	DHRS9	GATM	MYH11	FAM107A	NPTX2
DPCR1	DKK1	KLK1	PDGFRL	FCN1	PAX6
F5	ENO2	LEFTY1	RGS16	FOSB	PTGDS
FAM3D	IFI44L	LGALS2	SCUBE2	FPR1	QPCT
GPX2	IFIT1	MT1G	SFRP2	FYB	RAB26
LGALS4	IGF2BP3	PLA2G1B		GIMAP7	SCG5
MMP1	IRX3	PPY		GZMA	STMN2
NPC1L1	ISG15	PRSS3		GZMB	THBS4
PGC	KRT7	REG1A		HBB	ZBTB16
PIGR	LAMA3	SERPINA5		HLA-DQA1	
ST6GALNAC1	LAMB3	SLC30A2		IL1B	
TFF1	LAMC2	SLC3A1		IL33	
TFF2	MYEOV	TMED6		IL6	
TFF3	PHACTR3			IL7R	
VILL	PSCA			LTB	
VNN1	PTGS2			S100A8	
VSIG2	S100A4			SCARA5	
	SFN			SFRP1	
	SLC2A1			SLIT3	
	SPRR3			SPOCK2	
	UBE2C			SRGN	

### Functional annotation of PDAC subtypes

**Fig. 3:**
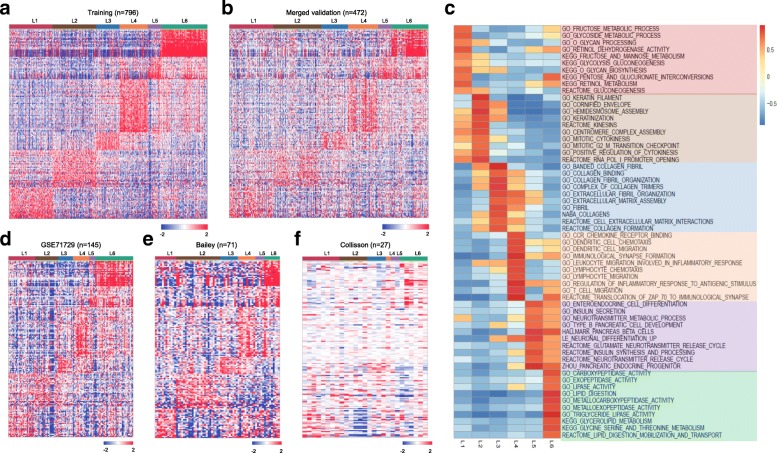
Functional annotation of PDAC subtypes. **a** Heatmap showing six subtypes of PDAC in training dataset using the 160 subtype specific genes, which reveals distinct gene expression patterns between subtypes. **b** Heatmap also showing six subtypes of PDAC in merged validation dataset using the 160 subtype specific genes, with similar gene expression patterns (subtype specific genes are highly expressed in the corresponded subtype) as observed in the training dataset. **c** GSEA analysis reveals distinct enriched gene sets between subtypes. In the heatmap, rows are defined by the selected 60 gene sets, and columns by consensus scores for each subtype. Subtype enriched gene sets are highlighted by different color, L1 (light red), L2 (light brown), L3 (light blue), L4 (light orange), L5 (light purple) and L6 (light green). **d**-**f** Heatmaps showing six subtypes of PDAC in three independent validation datasets (GSE71729, Bailey and Collisson) using the 160 subtype specific genes, with similar gene expression patterns (subtype specific genes are highly expressed in the corresponded subtype) as observed in the training dataset

There are distinct gene expression patterns between subtypes as observed in the heatmaps from both training and merged validation datasets (Fig. 3a-b). In the heatmap, columns correspond to PDAC patients, and rows to 160 genes. Gene expression matrices were median centered and expression values were represented by different colors, red means higher expression values, and green, lower ones. We have found that carbohydrate metabolism genes such as *ALDOB*, *CA2*, *NPC1L1* and *PGC* are highly expressed in L1. Cell proliferation and epithelium-associated genes, such as *CCNB2*, *CDKN2A*, *SFN*, *UBE2C*, *SPRR3*, *DHRS9* and *CRABP2* are enriched in L2 subtypes. *GREM1*, *MFAP5*, *COL12A1*, *COL10A1*, *COL8A1* and other collagen or ECM-related genes are upregulated in L3. Immune related genes such as *CCL*, *CCR7* and *CD* gene families are enriched in L4 subtype. Neuroendocrine-associated genes such as *PAX6*, *IAPP*, *G6PC2*, *ABCC8* and *ZBTB16* are highly expressed in L5. And lastly, *CLPS*, *PLA2G1B*, *CEL*, *ALB*, *CPA1*, *CPB1*, *CTRL*, *SLC3A1*, *PRSS3* and *ANPEP*, which are involved in lipid and protein metabolism, are enriched in the L6 subtype (Table 2 and Additional file 5: Figure S3).

To identify gene sets enriched in each subtype, we then performed GSEA analysis. GSEA is a widely used method to interpret expression data at the level of gene sets, or groups of genes that share a common biological function, or regulation [52]. We subsequently selected altogether 60 most representative gene sets for L1-L6 to build a pathway heatmap, which reveals distinct gene sets enriched in each subtype (Fig. 3c). Based on the biological functions of the selected gene sets, we further grouped the six-subtype into tumor-specific and stroma-specific subtypes. Tumor-specific subtypes include L1, L2 and L6, which are associated with cell proliferation and metabolism-related gene sets. Specifically, L1 has enriched carbohydrate metabolism-related gene sets. L2 is enriched for cell proliferation and epithelium-associated gene sets. And L6 is enriched for lipid and protein metabolism-related gene sets. Stroma-specific subtypes include L3, L4 and L5, which may contain high nonepithelial contents such as collagen, immune and islet cells, respectively. For instance, L3 is enriched for collagen and ECM related gene sets. L4 is enriched for various immune related gene sets. And L5 is enriched for neurotransmitter and insulin secretion related gene sets. Significantly enriched gene sets for each subtype were displayed in Additional file 6: Table S3.

### Clinical characterization of PDAC subtypes

**Fig. 4:**
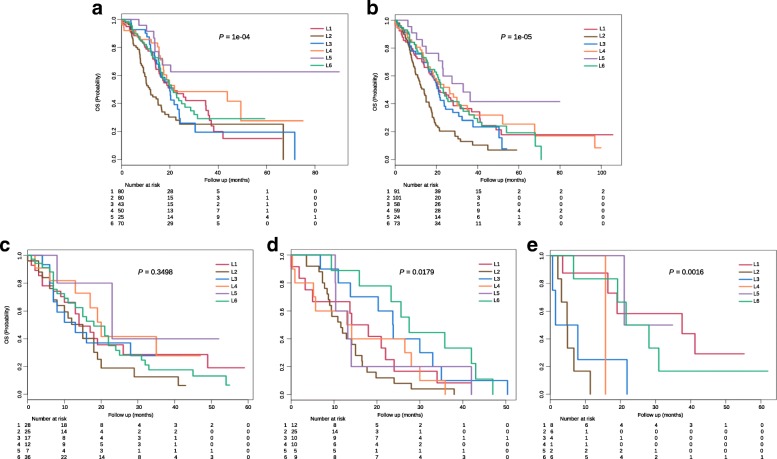
Clinical characterization of PDAC subtypes. **a** Kaplan-Meier survival curve comparing survival of L1 (red), L2 (brown), L3 (blue), L4 (orange), L5 (purple) and L6 (green) patients in the training dataset. Survival difference was tested using the log-rank test. *P*-values of less than 0.05 were considered statistically significant. **b** Kaplan-Meier survival curve in the merged validation dataset. **c**-**e** Kaplan-Meier survival curves in GSE71729, Bailey and Collisson datasets

About 348 patients (~ 43.7%) in the training dataset have clinical information. Their subtype labels and associated overall survival information were used to perform survival analysis and clinical characterizations. Kaplan-Meier analysis indicated that L2 has the worst clinical outcomes compared with other five subtypes (Fig. 4a). During the first 24 months after diagnosis, approximately 75% patients in L2 and L3, respectively, were censored. And the death rate in L2 was larger than that in L3, as observed in a steeper slope in the survival curves (Fig. 4a). Although there were no significant survival differences in L1, L3, L4 and L6 during the first 20 months after diagnosis, the survival differences were observed after 20 months, and the death rate of L3 and L6 rapidly increased compared with L1 and L4. L5 always has good clinical outcomes compared with the other 5 subtypes. We also observed a similar overall survival differences between subtypes in the merged validation dataset (Fig. 4b). Lastly, we did the Fisher’s exact test to investigate if the subtype memberships have any associations with other clinical factors, such as age, gender, race, tumor stage and grade. Results shows that only tumor grade have certain associations with subtypes (*p*-value < 0.01). For example, more than 97% patients in L2 and more than 95% patients in L3 have moderately or poorly differentiated tumor cells, whereas about 32% patients in L5 have well differentiated tumor cells (Additional file 2: Table S1). This analysis demonstrates that other clinical factors (such as age, gender, race and tumor stage) cannot predict overall survival, and supports the use of subtypes as a new and reliable prognostic factor in PDAC.

### Cross comparison of the identified subtypes with published studies

**Fig. 5:**
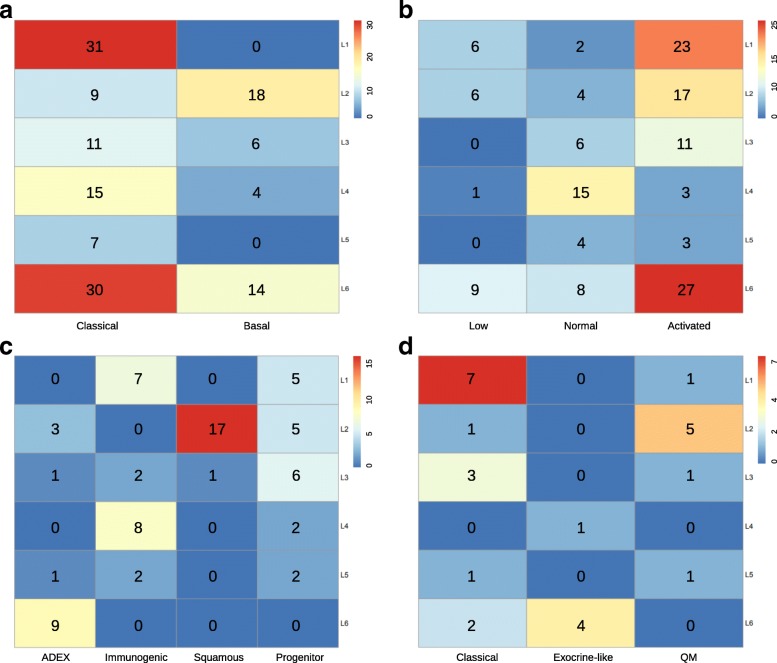
Cross comparison of identified subtypes with published results. We used our classifier and three published classifiers to classify PDAC patients, respectively, which produced two-dimensional matrices with rows correspond to our classification results and columns correspond to other classification results. **a** Contingency heatmap of GSE71729 dataset. Numbers in the heatmap represent patient numbers. Row labels: our classifier’s results, and column labels: GSE71729 stroma classifier’s results. **b** Contingency heatmap of GSE71729 dataset. Numbers in the heatmap represent patient numbers. Row labels: our classifier’s results, and column labels: GSE71729 tumor classifier’s results. **c** Contingency heatmap of Bailey dataset. Numbers in the heatmap represent patient numbers. Row labels: our classifier’s results, and column labels: Bailey classifier’s results. **d** Contingency heatmap of Collisson dataset. Numbers in the heatmap represent patient numbers. Row labels: our classifier’s results, and column labels: Collisson classifier’s classification results

To compare our classification system with three previously published results [18, 20, 23], we then used our PDAC classifier to classify these three cohorts, separately. Gene expression heatmaps (Fig. 3d-f) and survival curves (Fig. 4c-e) show much similar patterns between validation datasets and the training dataset, which indicate the existence of six subtypes in other cohorts as well. Although some inconsistent results exist, such as the log rank p-value was not significant in GSE71729 dataset, and the survival curves in all three datasets were not followed the exact patterns as observed in the training dataset. We believe such inconsistency were caused by the smaller sample size in the validation datasets (145 PDAC in GSE71729, 71 PDAC in Bailey and 27 PDAC in Collisson set), as compared with a larger cohort size in the training dataset (796 PDAC). The corresponding sample labels in these three datasets were downloaded from the published papers, contingency tables were subsequently built and visualized by heatmaps (Fig. 5a-d). L1 and L6 were much similar to the GSE71729’s classical subtype. L2 was close to the GSE71729’s basal subtype. L4 was resemble to the GSE71729’s normal subtype. L6, L1 and L2 were similar to the GSE71729’s activated subtype. In the Bailey dataset, L6 was similar to the ADEX subtype. L4 and L1 were close to the immunogenic subtype. L2 was resemble to the squamous subtype, and L3 was similar to the pancreatic progenitor subtype. Lastly, L1 and L3 were similar to the Collison’s classical subtype. L6 was close to the Collison’s exocrine-like subtype. L2 was related to the Collison’s quasi-mesenchymal subtype. All these similarities corresponded well with the molecular and clinical characteristics of the six subtypes identified in our study, which confirmed the correctness of the characteristics we found on these six subtypes.

## Discussion

Heterogeneity makes a cancer not just a single disease and this poses a significant challenge to the treatment of cancer patients. With the advent of genome-wide molecular profiling of cancers, especially the advancements in gene expression profiling technologies, researchers can depict genetic changes to better understand the heterogeneity of cancers. Compared with traditional classification of cancers, gene expression based classification can be used to classify cancers into subgroups with distinct molecular characteristics and clinical implications. Gene expression based classification of cancer was first proposed by Golub et al. [12]. The expression pattern of the 50 most informative genes was measured, and self-organizing maps (SOMs) clustering method was applied [53] to classify 38 leukemia patients into two prognostic groups without previous knowledge of these classes. This demonstrated the fidelity of cancer classification based solely on gene expression patterns [12]. In our study, we applied NMF to perform gene expression based classification of PDAC. We identified six molecular and clinical distinct subtypes, which not only proved that PDAC is a highly heterogeneous disease, but also demonstrated that gene expression based classification of cancer is molecular and clinical significant.

The identification of cancer subtypes can be difficult due to the lack of tumor samples available for study. The majority of PDAC patients (~ 80%) were first diagnosed with advanced tumor stages and were not suitable for resection. Some studies have overcome this problem by combining different sources of samples into their studies to increase the sample size [18, 54, 55]. Concatenating different datasets into a single dataset can be both significant and challenging. On one hand, integrating samples from various of independent studies can increase the statistical power and robustness. On the other hand, there exist batch effects or called non-biological differences between these datasets. Luckily, methods like Empirical Bayes (EB) [56], Surrogate Variable Analysis (SVA) [57] or Distance Weighted Discrimination (DWD) [58] can be used to remove such batch effects. For example, TCGA’s glioblastoma (GBM) subtyping study [59] integrated gene expression data from 200 GBM assayed on three platforms (Affymetrix HuEx array, Affymetrix U133A array and Agilent 244 K array) into a single dataset. Factor analysis and consensus hierarchical clustering [60] were subsequently performed for feature selection and cluster identification, respectively. The above work also used an independent dataset which contains 260 GBMs from four previously published datasets as validation dataset, and subtypes were predicted using 840 gene expression profiles and ClaNC (a nearest centroid-based classifier) [61]. In a recent publication of diffuse glioma subtyping study from TCGA [62], the authors used ComBat batch effect removal method [45] to combine multi-platform and multi-tumor mRNA expression data.

Using different patient cohorts, gene expression platforms and clustering methods can produce totally different classification results. For example, epithelial ovarian cancer (EOC) has been classified into 4 to 6 subtypes [63–65], colorectal cancer (CRC) 3 to 6 subtypes [66], and PDAC 2 to 4 subtypes were identified by different research groups [18–23]. Thus, integrating multiple patient cohorts to reduce the racial/ethnic and platforms differences, together with a unified clustering method for classification is necessary and important. In our study, in order to build a generalizable classification model for PDAC, we combined multiple PDAC gene expression datasets, and adjusted the introduced batch effect using ComBat. Our study involves more than 1200 PDAC patients, therefore, the statistical power was significantly increased. We have several advantages compared with previous studies [18–23], such as we identified novel subtypes in PDAC, we used NMF biclustering method to extract features which are more subtype specific, and finally we built a deep learning-based classification system for PDAC which can be used to classify new patients.

The expression profiling of the 160 genes identified from our study can stratify PDAC patients into six subtypes. And each subtype is characterized by the expression of a subset of genes which sharing similar biological functions, respectively. For example, L1 and L6 subtypes have enriched with metabolism-related genes; L2 and L3 have enriched with epithelium-associated and ECM-related genes, respectively; immune response genes in L4, and neuroendocrine related genes in L5. These specific expression profiles can be used to predict the clinical outcomes for each subtype, such as epithelium and cell proliferation gene profiles in L2 are related with poor prognosis; metabolism and ECM profiles in L1, L6 and L3 are associated with intermediate survival; and immune and neuroendocrine-associated profiles in L4 and L5 are correlated with relatively good clinical outcomes. The subtyping results from our study can also be interpreted at the cellular level. Low tumor cellularity and the presence of abundant stroma intermixed with normal cells are the common features of PDAC [20, 24]. Although microdissection can be used to enrich tumor cells, non-tumor components still account for a significant proportion in PDAC tissue biopsies. For example, stroma comprises on average 48% of Moffitt et al. [20] primary tumor samples with a standard deviation of 30%; and in the TCGA’s samples [24], the tumor purity ranged from 0 to 53% (median 18%). Current tissue-level expression profiling technologies process thousands of tumor and non-tumor cells at the same time, so differences or heterogeneity between patients may also result from changes in the proportions of cell types in samples. If so, then what machine learning models learned from bulk data is the cell-type proportions among samples, which can be benefit from a large group of patient’s data. Perhaps cell-type proportions are really informative, which have important implications in the treatment strategies for cancer patients. In our study, epithelial cells concentration in tumor-specific subtypes (L1, L2 and L6) may greater than the stroma-specific subtypes (L3, L4 and L5), which may suggest that L3, L4 and L5 should be treated differently from L1, L2 and L6. For instance, malignant epithelial cells in L2 may account for the largest proportion, thus more intensive treatments should be considered for L2 patients. L1 and L6, two metabolism-related subtypes may be treated by some metabolic drugs [67]. Furthermore, collagen-targeted therapies for L2, immunotherapies for L4, and endocrine cell therapies for L5.

## Conclusions

In summary, we have identified six biologically informative subtypes of PDAC, which corresponding well with their molecular features and clinical outcomes. The 160 subtype specific biomarkers and the deep learning model have the potential to drive personalized therapies [68] and risk prediction [69] for the PDAC patients.

## Additional files

Additional file 1: **Figure S1.** PCA before and after batch effect correction for training and validation datasets via ComBat. (a) PCA on training dataset (*n* = 796) prior to batch effect correction. (b) PCA on training dataset (*n* = 796) after batch effect correction. (c) PCA on validation dataset (*n* = 472) prior to batch effect correction. (b) PCA on validation dataset (*n* = 472) after batch effect correction. (PDF 157 kb)

Additional file 2: **Table S1.** Clinical data with patient characteristics and statistical associations of six subtypes with clinical outcome. (DOCX 17 kb)

Additional file 3: **Figure S2.** Heatmap of consensus matrices from 30 runs for each rank (2 to 10) on the training dataset. (PDF 12 kb)

Additional file 4: **Table S2.** Confusion Matrices in internal training and validation sets. (XLS 25 kb)

Additional file 5: **Figure S3.** Boxplots showing mean gene expression patterns of some interesting biomarkers between six subtypes (L1 gene list: *ALDOB*, *CA2*, *NPC1L1* and *PGC*. L2 gene list: *CCNB2*, *CDKN2A*, *SFN*, *UBE2C*, *SPRR3*, *DHRS9* and *CRABP2*. L3 gene list: *GREM1*, *MFAP5*, *COL12A1*, *COL10A1* and *COL8A1*. L4 gene list: *CCL*, *CCR7* and *CD* gene families. L5 gene list: *PAX6*, *IAPP*, *G6PC2*, *ABCC8* and *ZBTB16*. L6 gene list: *CLPS*, *PLA2G1B*, *CEL*, *ALB*, *CPA1*, *CPB1*, *CTRL*, *SLC3A1*, *PRSS3* and *ANPEP*). X-axis: six subtypes, y-axis: gene expression values. Paired t-test was used to determine whether there were statistically significant differences in mean gene expression between subtypes, results show that all six comparisons are significant (*p*-value < 2.2e-16). (PDF 62 kb)

Additional file 6: **Table S3.** Significantly enriched gene sets for each subtype. (XLS 883 kb)

## Abbreviations

cpm: Counts per million
CRC: Colorectal cancer
DWD: Distance Weighted Discrimination
EB: Empirical Bayes
ECM: Extracellular matrix
EOC: Epithelial ovarian cancer
GBM: Glioblastoma
GEO: Gene Expression Omnibus
GSEA: Gene set enrichment analysis
ICGC: International Cancer Genome Consortium
MAD: Median absolute deviation
NMF: Non-negative matrix factorization
PDAC: Pancreatic ductal adenocarcinoma
SAM: Significance Analysis of Microarrays
SVA: Surrogate Variable Analysis
SVD: Singular value decomposition
TCGA: The Cancer Genome Atlas
TMM: Trimmed mean of M-values
