# Supplementary figures and images for: Gene expression profiling of 1200 pancreatic ductal adenocarcinoma reveals novel subtypes
Source: BMC Cancer. 2018 May 29;18:603. doi: 10.1186/s12885-018-4546-8 (PMC5975421; doi:10.1186/s12885-018-4546-8)

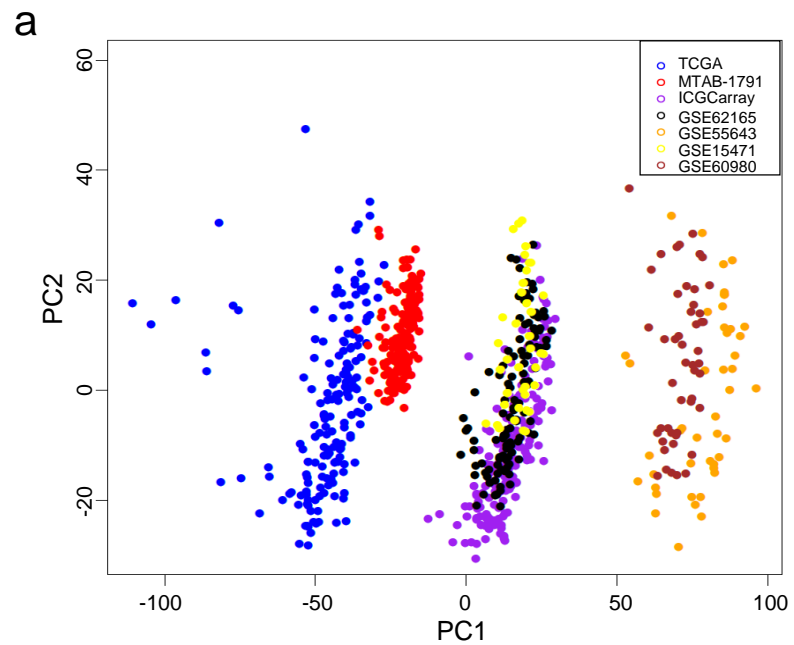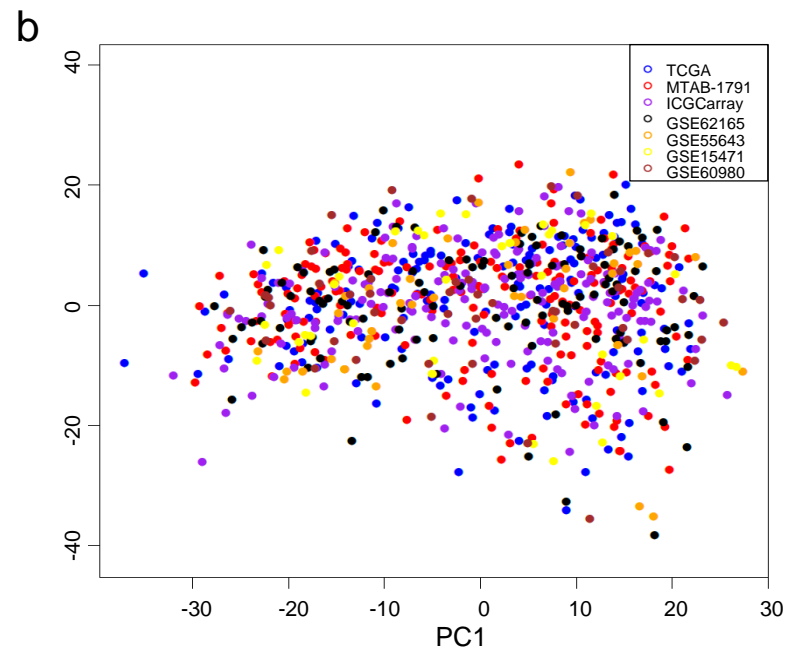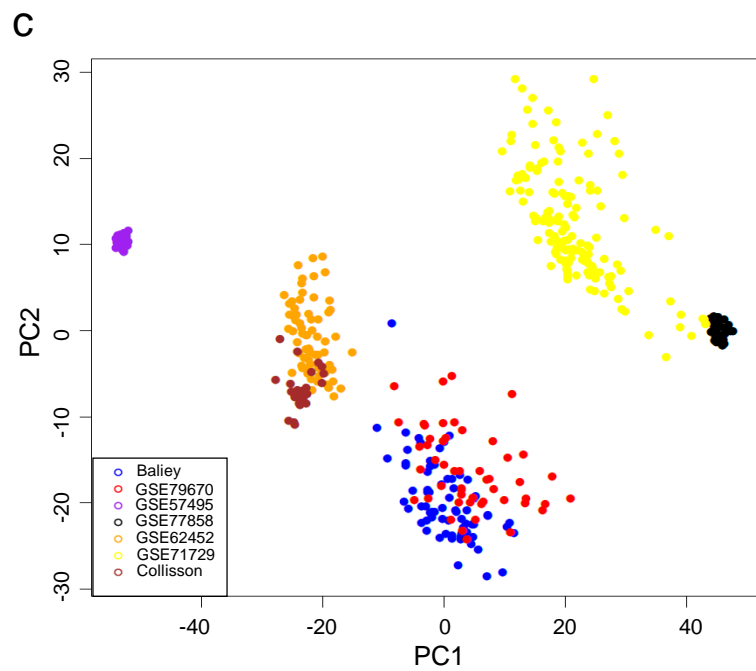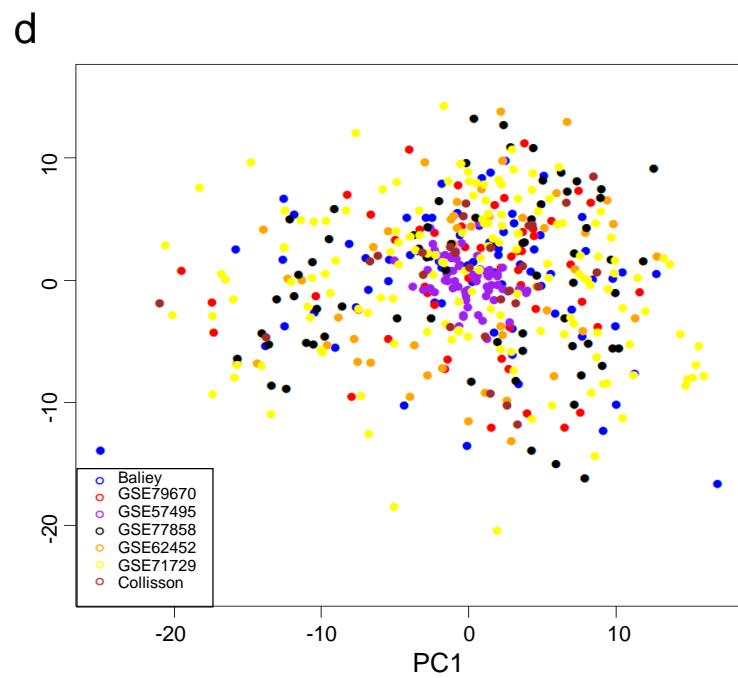

Supplement: Supplementary file 1 — Figure S1. PCA before and after batch effect correction for training and validation datasets via ComBat. (a) PCA on training dataset (n = 796) prior to batch effect correction. (b) PCA on training dataset (n = 796) after batch effect correction. (c) PCA on validation dataset (n = 472) prior to batch effect correction. (b) PCA on validation dataset (n = 472) after batch effect correction. (PDF 157 kb) [file 12885_2018_4546_MOESM1_ESM.pdf]

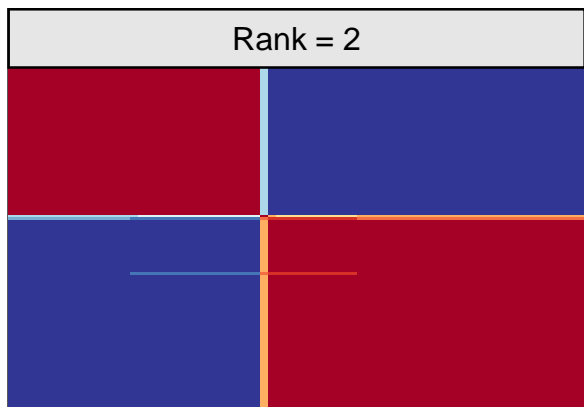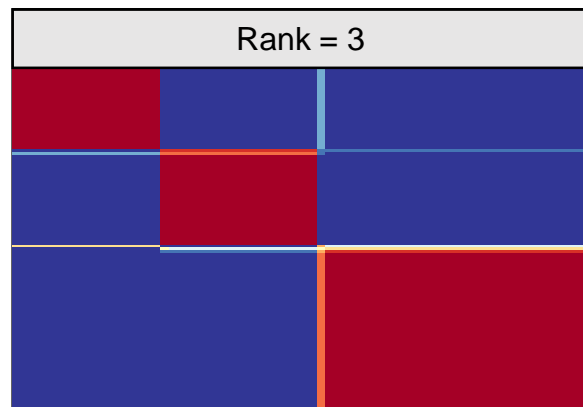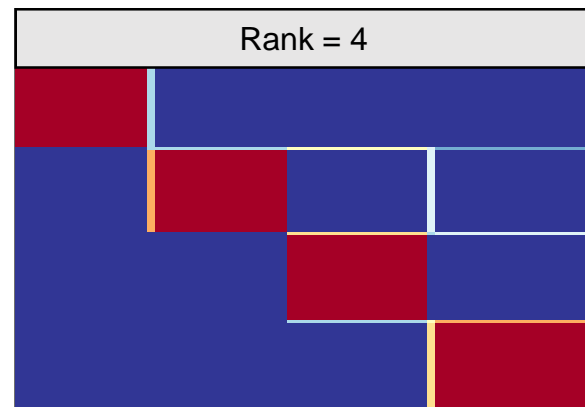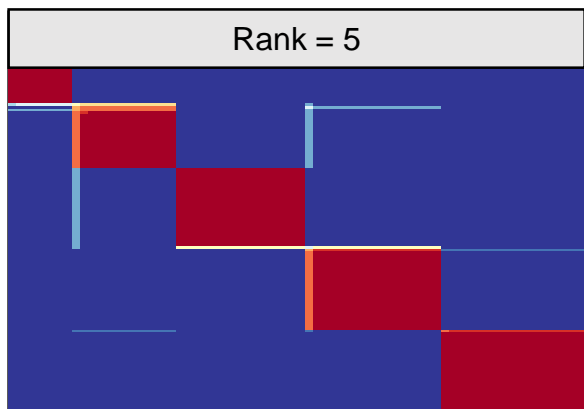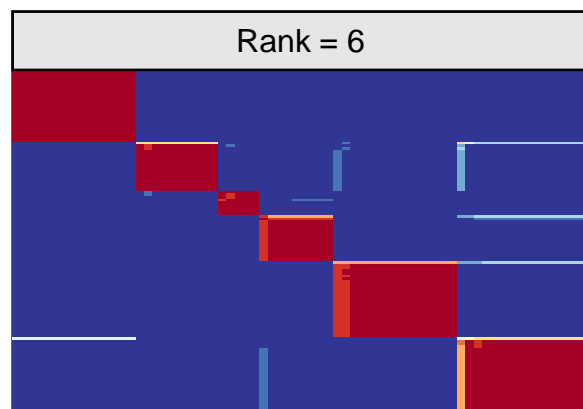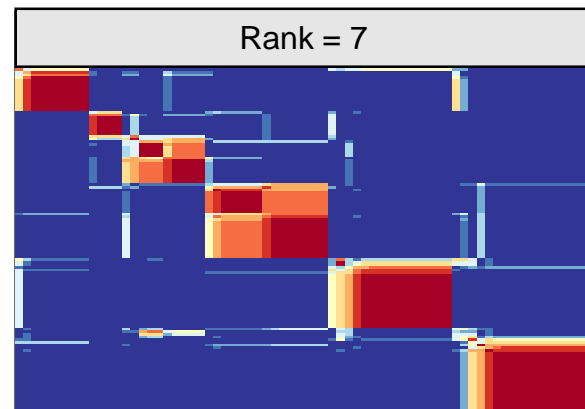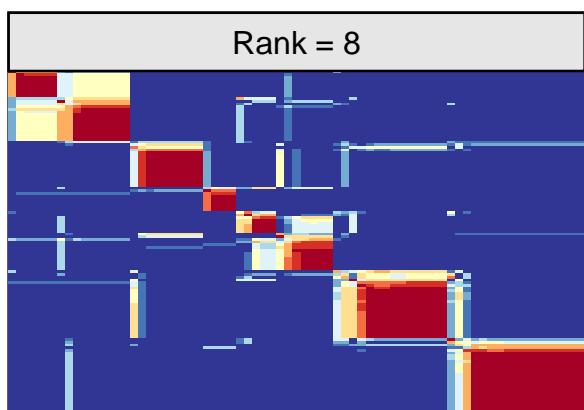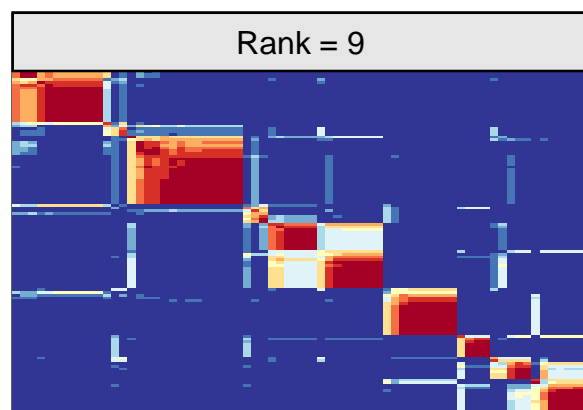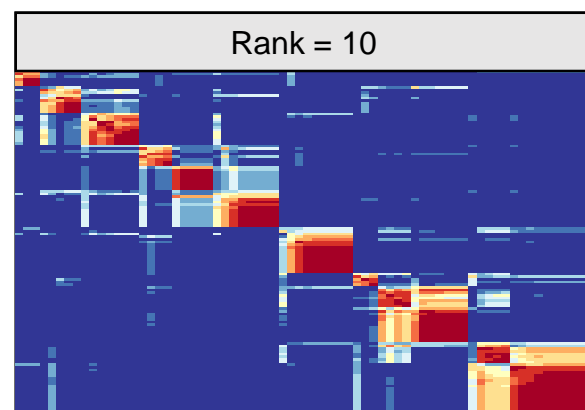

Supplement: Supplementary file 3 — Figure S2. Heatmap of consensus matrices from 30 runs for each rank (2 to 10) on the training dataset. (PDF 12 kb) [file 12885_2018_4546_MOESM3_ESM.pdf]

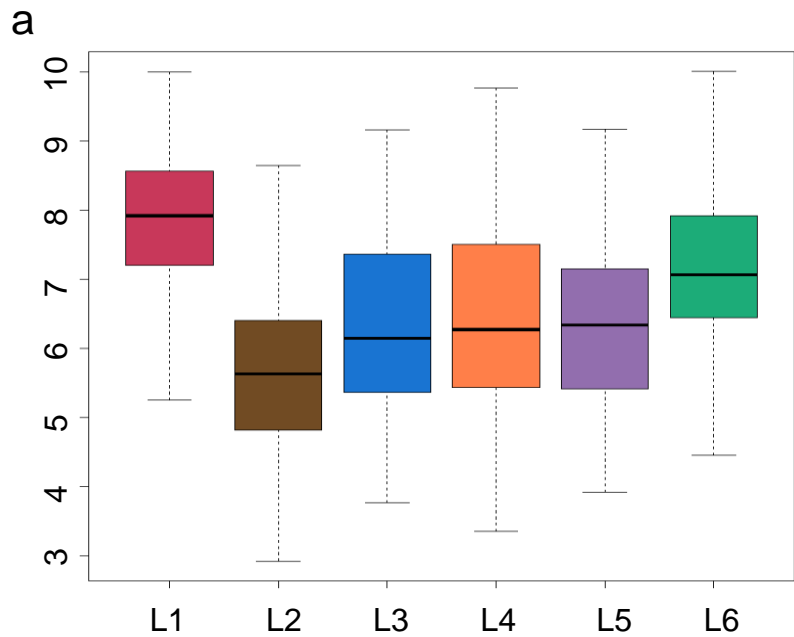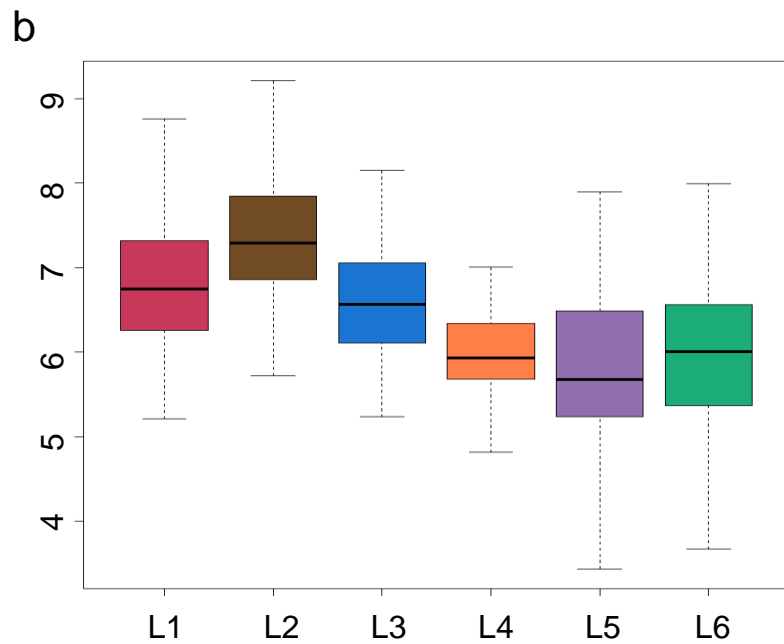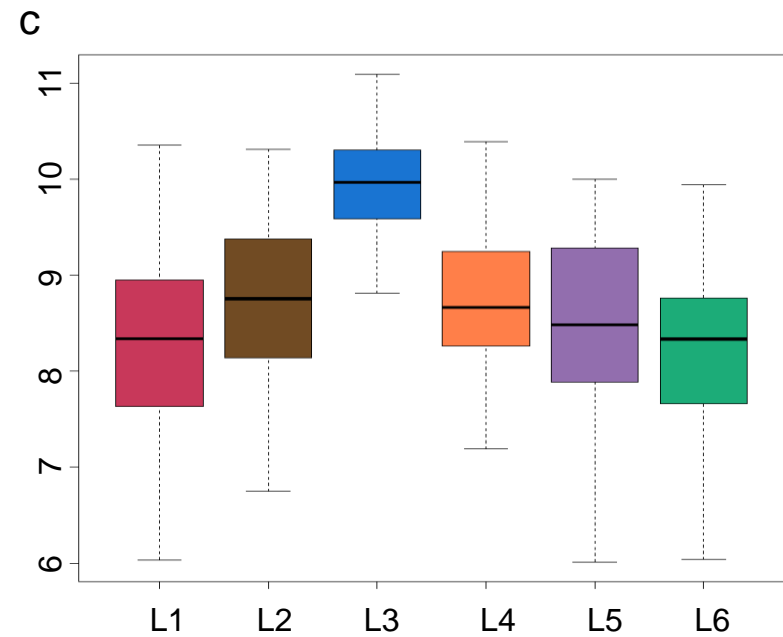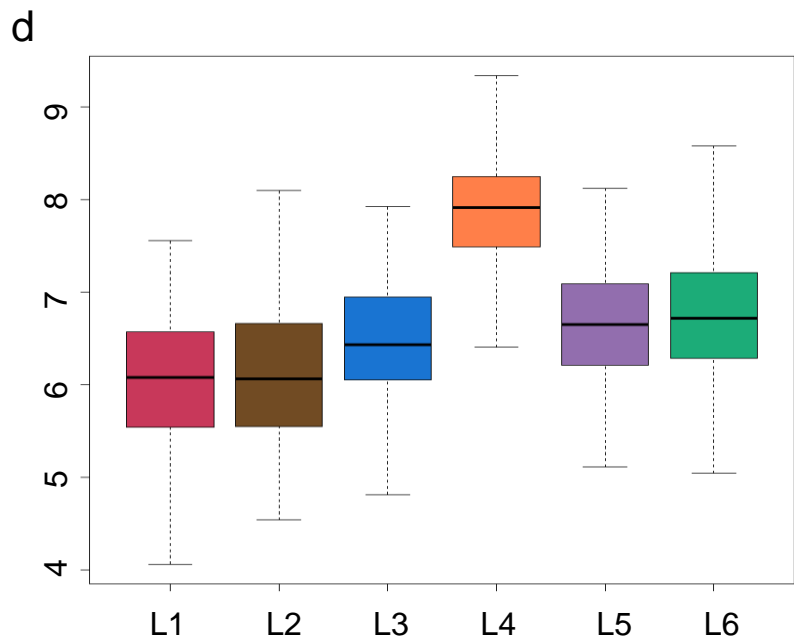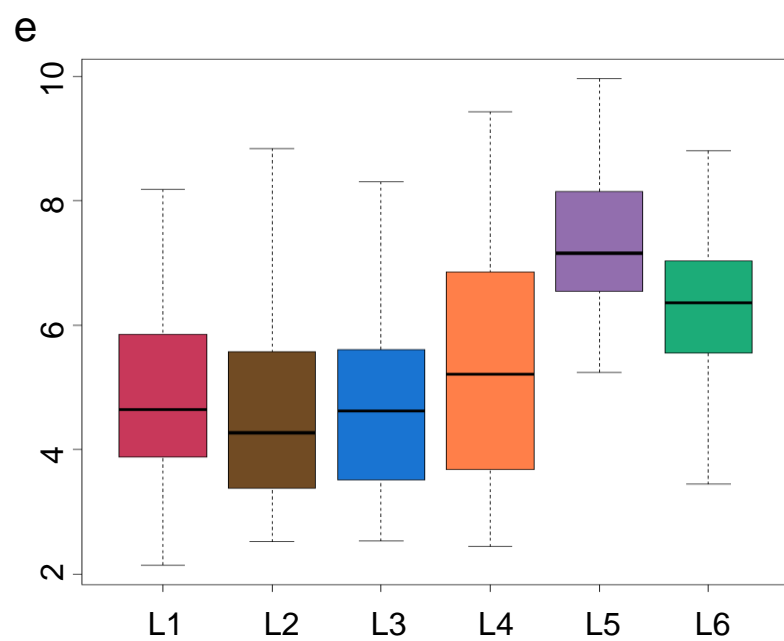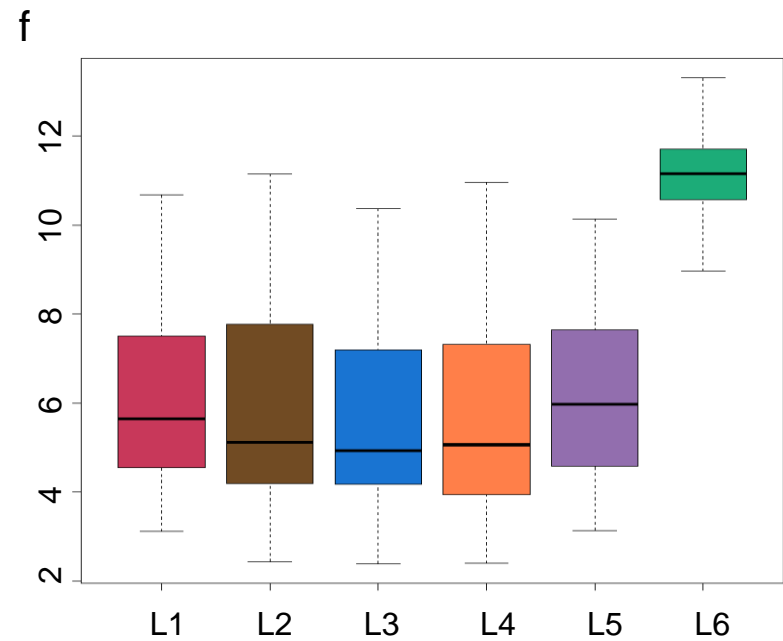

Supplement: Supplementary file 5 — Figure S3. Boxplots showing mean gene expression patterns of some interesting biomarkers between six subtypes (L1 gene list: ALDOB, CA2, NPC1L1 and PGC. L2 gene list: CCNB2, CDKN2A, SFN, UBE2C, SPRR3, DHRS9 and CRABP2. L3 gene list: GREM1, MFAP5, COL12A1, COL10A1 and COL8A1. L4 gene list: CCL, CCR7 and CD gene families. L5 gene list: PAX6, IAPP, G6PC2, ABCC8 and ZBTB16. L6 gene list: CLPS, PLA2G1B, CEL, ALB, CPA1, CPB1, CTRL, SLC3A1, PRSS3 and ANPEP). X-axis: six subtypes, y-axis: gene expression values. Paired t-test was used to determine whether there were statistically significant differences in mean gene expression between subtypes, results show that all six comparisons are significant (p-value < 2.2e-16). (PDF 62 kb) [file 12885_2018_4546_MOESM5_ESM.pdf]
