# Supplementary material for: Gene expression profiling of 1200 pancreatic ductal adenocarcinoma reveals novel subtypes
Source: BMC Cancer. 2018 May 29;18:603. doi: 10.1186/s12885-018-4546-8 (PMC5975421; doi:10.1186/s12885-018-4546-8)
Supplement: Supplementary file 2 — Table S1. Clinical data with patient characteristics and statistical associations of six subtypes with clinical outcome. (DOCX 17 kb) [file 12885_2018_4546_MOESM2_ESM.docx]

**Supplemental Table 1. Clinical data with patient characteristics and statistical associations of six subtypes with clinical outcome.**

|  | **Training set (n=796, 348 have clinical information)** | | | | | | | **Combined validation set (n =472, 406 have clinical information)** | | | | | | |
| --- | --- | --- | --- | --- | --- | --- | --- | --- | --- | --- | --- | --- | --- | --- |
|  | L1 (n=80) | L2 (n=80) | L3 (n=43) | L4 (n=50) | L5 (n=25) | L6 (n=70) | p-value | L1 (n=91) | L2 (n=101) | L3 (n=58) | L4 (n=59) | L5 (n=24) | L6 (n=73) | p-value |
| Age, years | 66.22 | 65.02 | 65.14 | 67.16 | 65.08 | 64.78 | 0.24 | 65.71 | 65.37 | 63.76 | 67.05 | 63 | 67.89 | 0.09 |
| Sex, male percentage | 47/80 (59%) | 45/80 (56%) | 28/43 (65%) | 24/50 (48%) | 13/25 (52%) | 35/70 (50%) | 0.57 | 19/27 (70%) | 23/39 (59%) | 9/16 (56%) | 9/19 (47%) | 3/7 (43%) | 6/14 (43%) | 0.22 |
| Tumor Grade |  |  |  |  |  |  | 5e-4 |  |  |  |  |  |  | 5e-4 |
| 1 - Well differentiated | 10 | 1 | 2 | 7 | 8 | 12 |  | 2 | 0 | 1 | 1 | 2 | 1 |  |
| 2 - Moderately differentiated | 50 | 38 | 27 | 36 | 12 | 40 |  | 25 | 12 | 11 | 12 | 6 | 23 |  |
| 3 - Poorly differentiated | 19 | 39 | 14 | 7 | 5 | 17 |  | 8 | 35 | 7 | 9 | 3 | 6 |  |
| 4 – Undifferentiated | 1 | 1 | 0 | 0 | 0 | 1 |  | 0 | 0 | 1 | 0 | 0 | 0 |  |
| NA | 0 | 1 | 0 | 0 | 0 | 0 |  | 69 | 67 | 43 | 50 | 21 | 57 |  |
| TNM stage |  |  |  |  |  |  | 0.09 |  |  |  |  |  |  | 0.56 |
| I | 6 | 6 | 2 | 4 | 8 | 4 |  | 2 | 7 | 5 | 4 | 2 | 5 |  |
| II | 72 | 70 | 37 | 44 | 15 | 65 |  | 32 | 50 | 21 | 28 | 14 | 31 |  |
| III | 1 | 2 | 1 | 1 | 0 | 1 |  | 5 | 4 | 1 | 4 | 0 | 1 |  |
| IV | 1 | 2 | 3 | 1 | 0 | 0 |  | 1 | 5 | 1 | 0 | 0 | 5 |  |
| NA | 0 | 0 | 0 | 0 | 2 | 0 |  | 64 | 48 | 35 | 36 | 16 | 45 |  |
| Race |  |  |  |  |  |  | 0.14 |  |  |  |  |  |  | 0.36 |
| Asian | 8 | 5 | 6 | 2 | 1 | 2 |  | 1 | 4 | 1 | 0 | 1 | 0 |  |
| White | 66 | 71 | 36 | 46 | 24 | 62 |  | 24 | 33 | 14 | 16 | 6 | 12 |  |
| African | 4 | 1 | 0 | 1 | 0 | 3 |  | 2 | 1 | 1 | 1 | 0 | 2 |  |
| NA | 2 | 3 | 1 | 1 | 0 | 3 |  | 77 | 76 | 47 | 55 | 25 | 73 |  |
